# Observations on a Case of Paralysis of the Extensor Muscles of the Hand and Fingers, Accompanied with a Separation of the Bones of the Metacarpus

**Published:** 1800-03

**Authors:** 

**Affiliations:** Physician at Bagniéres


### *Observations on a Case of Paralysis of the Extensor Muscles of the Hand and Fingers, accompanied with a Separation of the Bones of the Metacarpus:* by Cit. Bourguet, Physician at Bagniéres.

[Extracted from the Recueil Periodique, No. XXXIV.]

CORN. ———, an inhabitant of Madrid, came to Bagniéres afflicted with the following disease: He could not hold up nor extend his hands, but they continually hung down as if deprived of animation. His fingers were paralytic, but his thumbs were not affected; he could extend the former but not bend them; the lower parts of his arms were in a state of atrophy, their external surface was particularly flabby; the thenar eminence of each hand was in a similar state, and the patient was very unhappy.

The cause of this disease was attributed to the veneral virus, and under this idea the patient had submitted a long time to various modes of treatment; but, whenever he experienced any relief, it was from the use of the tonic baths of the place in which I reside. His disease returning in the present year, (the 5th of the Republic) he resorted to Bagniéres, and took the waters without the least effect: he then consulted me; but not being satisfied with his statement of the case, I examined him more attentively, and found the extensor muscles of his wrists and fingers in a paralytic state; the epidermis cold and almost insensible; the extensor muscles of the thumbs alone retained their energy. The joints of the wrists and fingers were always contracted, and those of the thumbs very weak, so that the paralytic yielding to the efforts of the sound parts, the hands remained constantly bent, and great exertion was necessary to extend them. All the bones of the wrist were separated. Between the articulations was a discharge of thick synovia; the soaked ligaments had lost their tone, and those bones could be moved separately without occasioning pain. The sheaths of the tendons were likewise soaked with synovia; the cellular membrane was relaxed and tumefied, so that the hands were larger and heavier than in their natural state. Having well considered these circumstances, I could not ascribe them them to the exiſtence of a venereal virus; beſides which, I was too well aware of the effects of mercury on the minor articulations, to apply that medicine. I conſequently purſued the following mode of treatment: I firſt applied to the diſeaſed part a proper bandage, provided with ſheaths, to relieve the extenſor muſcles from the twitching to which they were ſubject, to aid the re-abſorption of the ejected fluids, and to promote the approximation of the bones of the carpus. During this time, aromatic, ſpirituous, and ſtimulant fumigations, applied to the extenſors alone, and frictions of the ſame kind, in a ſmall degree reduced them towards their natural ſtate. I then directed, round the metacarpus, a plaſter of gum ammoniac diſſolved in vinegar of ſquills; I ſeconded the effect which it produced, by the uſe of tonic fomentations, always applied to the extenſors. I ſoon had the pleaſure of obſerving muſcular motion re-appear; the bones of the metacarpus united, and the articulation become more firm. I left off the rolled bandage, and made two leather gauntlets ſtrengthened with ſmall pieces of whalebone, to relieve the wriſts. I continued to employ diſſolvent and tonic remedies, and ordered the patient to drink of the warm diuretic waters of Barèges. He ſoon after departed for Paris. I adviſed him to make trial, as he paſſed, of the mineral waters of Dax. On his arrival at Paris, he procured, according to the plan I had given him, a ſilver inſtrument, which anſwered the purpoſe I expected from the uſe of the leather gauntlet. Some time after he returned to Bagnières, having now the uſe of his hands and wriſts, without the aid of any inſtrument; and at length he was perfectly cured.

---

### *Reflections on the preceding Obſervations:* *By* Cit. Botentuit.

Although the author may have followed ſuch indications as naturally occurred in the treatment of this diſeaſe, the ſucceſsful termination of which appears to be chiefly owing to the means employed, his ſtatement preſents details which have ſuggeſted to me the following reflections:

Firſt, he ſtates that he endeavoured to direct the action of aromatic fumigations on the extenſors alone.—But could he prevent their action from being communicated to the other muſcles? This appears to me to be problematical, conſidering the form of the medicine. We are indebted to Mr. Winſlow for an obſervation, in which the joint application of emollients and tonics relieved the antagoniſt muſcles, ſome of them being contracted, contracted, and others relaxed; but the action of thefe medicaments being directed to both fides of the neck, their feparate application became more eafy. Befides, Cit. Bourguet had nothing to fear from the action of tonics on the flexors, which were not difeafed, and which only kept the fingers bent, becaufe their antagonifts could not counter-balance their operation.

In applying a rolled bandage and fheaths, the author fays it was his intention to relieve the extenfor mufcles from the concomitant twitchings; but will the permanent contraction of a mufcle caufe its antagonift to experience a twitching, particularly when the latter is paralytic? When a mufcle does not contract, is it not in a ftate of relaxation? And can it be faid in the prefent cafe, let it fhoot, as long as the fibres are not carried beyond the point to which they ufually extend? It appears to me, that if the fheaths could then be ufeful, it was by preventing the joints from becoming gradually accuftomed to their ftate of contraction, fo as to admit no longer of the extenfion of the fingers.

The fecond intention of the author in the application of the bandage, was, he fays, to favour the re-abforption of the ejected fluids.

I doubt whether he has been able to obtain this advantage. I am well aware that a tight bandage, and a fkin prepared and laced on an extremity of the body, may reftore its elafticity, as well as that of the cellular texture, by oppofing in the firft place the humours in one part, difperfing the tumefactions, &c. but, in the prefent cafe, where the thick fynovia, according to Cit. Bourguet, occurred between the bones of the metacarpus, it appears to me that the remedies applied, could not produce the defired effect. In fhort, I fubmit my ideas to the decifion of the Society; as I have not had an opportunity of feeing cafes analogous to that which forms the fubject of this paper: but I have met with two where the fingers were bent in a fimilar manner, not by a palfy of the extenfors, but by the contraction of the flexor mufcles.

A young man, about twenty years of age, wounded himfelf in the hand with a fpit, (I do not know how deep) with which he was about to fpit a fowl. He experienced very little pain, no fymptoms occurred, and the wound was cured in a few days; but the fingers became bent by degrees, the hand totally clofed, and every time he attempted to open it, the pain became fo acute, that the patient was deprived of his fenfes. Emollients of every kind, and in every poffible form, were applied for a confiderable length of time, and the cure was effected at the end of fix months only.

A child, ten years of age, was thrown down in the ftreet, and and fell upon its open hand; the pain was but slight, and he passed the night quietly; but the next morning the fingers were entirely contracted. They could not be extended without force, and the child expressed great pain by his cries. Emollients in the form of baths, affusions, and fomentations, were applied, but without effect. I attempted, like Cit. Bourguet, to keep the fingers extended with sheaths, which caused much pain; and as soon as they were laid aside, the fingers again became contracted. I then resorted to my former methods, but with no better success. I tried to make alternate motions of flexion and extension, which were continued for several minutes, notwithstanding the cries of the child. After an intermission of a few seconds, I again moved the fingers, and observed that they became gradually less sensible of pain. The child's mother continued these motions during the day; and two days afterwards, the little patient recovered the perfect use of his fingers.

I have frequently seen, after fractures near the articulations, and even after reduced luxations, all the muscles of the wounded extremity in a semi-paralytic state. I have often succeeded by the means before mentioned, but not without inducing greak weakness, which remained in those parts for a considerable time. Too often, also, the patients continued lame, notwithstanding all the aid of art. I have several times advised persons afflicted with such complaints, to resort to the mineral springs, as their last resource; and particularly with the hope, that Time and Nature would accomplish the cure.

